# Intermittent hypoxia-induced METTL3 downregulation facilitates MGLL-mediated lipolysis of adipocytes in OSAS

**DOI:** 10.1038/s41420-022-01149-4

**Published:** 2022-08-06

**Authors:** Xiuji Huang, Xuming Huang, Haiyan Guo, Jin Li, Chunxia Zhou, Yuanli Huang, Chunliu Lai, Wan Zeng, Xiaozhen Tan, Lihong Niu, Hui Li, Jian Qi, Canmao Xie

**Affiliations:** 1 Department of Respiratory and Critical Care Medicine, The Seventh Affiliated Hospital, Sun Yat-sen University, Shenzhen, 518107 P.R. China; 2 Department of Thyroid and Vascular Surgery, Maoming People’s Hospital, Southern Medical University, Maoming, 525000 P.R. China; 3 Department of Gastroenterology, The Seventh Affiliated Hospital, Sun Yat-sen University, Shenzhen, 518107 P.R. China

**Keywords:** Diabetes, Fat metabolism, Respiratory tract diseases

## Abstract

Intermittent hypoxia (IH) is the core pathological feature of obstructive sleep apnea syndrome (OSAS), and insulin resistance (IR) is the most common metabolic complication of OSAS. Studies have shown that the levels of free fatty acids (FFAs), which are mainly released from adipocytes by lipolysis, are elevated in OSAS and play an important role in the development of IR. However, whether and how IH regulates adipocyte lipolysis in OSAS is not clear. Here, we revealed that the apnea hypopnea index was positively correlated with the serum levels of FFAs and FFA release from adipocytes in OSAS. In addition, IH facilitated lipolysis and FFA release from adipocytes by downregulating the level of METTL3. METTL3 downregulation impaired N6-methyladenosine (m6A) levels in MGLL mRNA and reduced MGLL expression, thereby promoting lipolysis. In addition, we identified YTHDF2 as the m6A reader that interacts with MGLL mRNA, accelerating its degradation. Furthermore, our data showed reduced levels of METTL3 and elevated levels of MGLL in the adipose tissues of OSAS patients and indicated an effect of METTL3 on lowering FFA levels and improving IR in rats with chronic IH. In conclusion, our study provides new insights into the development and treatment of IR in OSAS.

## Introduction

Obstructive sleep apnea syndrome (OSAS) is characterized by chronic intermittent hypoxia (IH) and sleep fragmentation and is the most common sleep respiratory disease in the clinic [1]. OSAS is often accompanied by various metabolic abnormalities that have a great impact on patient health [2]. It was reported that up to 50% of OSAS patients have metabolic abnormalities, and insulin resistance (IR) is the most common abnormality [2]. IR is an important pathophysiological feature of type 2 diabetes and is closely related to metabolic syndrome and cardiovascular complications in OSAS [3]. In recent years, studies have shown that IR is closely related to IH in OSAS. IH leads to abnormal IR and glucose metabolism both in mice and human beings [4, 5]. In addition, the use of continuous positive pressure ventilation (CPAP) to treat IH can improve the level of IR in patients with OSAS [6]. However, the underlying mechanism by which IH participates in the occurrence of IR in OSAS is not clear.

Free fatty acids (FFAs) are long-chain fatty acids that are mainly produced by adipocyte lipolysis [7]. During lipolysis, triglycerides are hydrolyzed by the adipose triglyceride lipase (ATGL), hormone-sensitive lipase (HSL) and monoacylglycerol lipase (MGLL) resulting in the release of FFAs and glycerol [8]. The released FFAs are transported to target organs, such as skeletal muscle and the liver, to participate in metabolic activities [9]. FFAs are key factors in the occurrence and development of IR and type 2 diabetes [10]. FFAs induce IR by inhibiting glucose utilization in muscle, the liver and other organs to reduce insulin sensitivity [11]. Importantly, studies have shown that the serum levels of FFAs in OSAS patients are significantly elevated [12]. Moreover, improving IH with CPAP treatment reduced the serum levels of FFAs in OSAS patients [13]. Whether and how IH influences lipolysis and FFA release from adipocytes in OSAS remains unclear.

RNA modification is an important type of epigenetic modification that regulates gene expression [14]. Among them, N6-methyladenosine (m6A) is the most abundant modification and plays a key role in the regulation of cellular functions [15]. m6A is written by the methyltransferase complex and recognized by methylation recognition enzymes, including YTH domain containing protein 1/2 (YTHDC1/2) and YTH N6-methyladenosine RNA binding protein 1/2/3 (YTHDF1/2/3), which regulate the stability, translation efficiency, splicing and other characteristics of RNA [16]. Methyltransferase 3, N6-adenosine-methyltransferase complex catalytic subunit (METTL3) is the catalytic subunit and core part of the methyltransferase complex. Studies have shown that METTL3 participates in various pathophysiological processes, including glucose homeostasis and lipid metabolism. However, the role of METTL3 and m6A in IH and lipolysis is still unknown.

In our study, we found that IH was closely related to FFA release in OSAS and facilitated lipolysis and FFA release from adipocytes by reducing the level of METTL3. We further revealed that MGLL was the m6A modification target of METTL3 and identified the related m6A reader YTHDF2. In addition, our data showed the effect of METTL3 on lowering FFA levels and improving IR in rats with chronic intermittent hypoxia (CIH), which provides potential therapeutic targets for treating IR in OSAS.

## Results

### IH stimulation promoted lipolysis in adipocytes

**Fig. 1:**
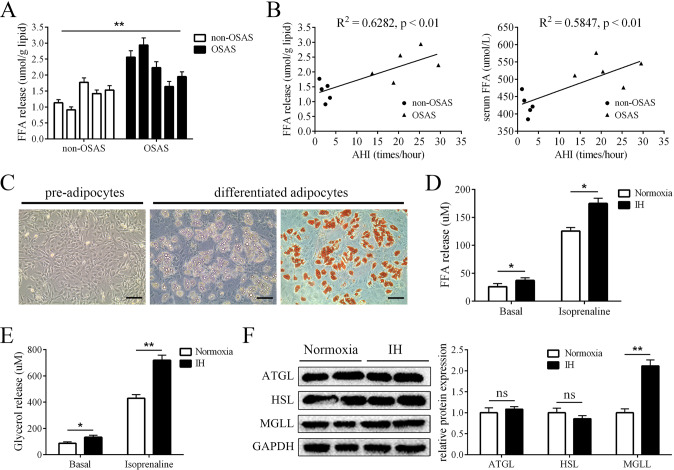
IH stimulation promoted lipolysis in adipocytes. **A** The levels of FFA release of lipids from OSAS and non-OSAS patients, *n* = 5. **B** The AHI was positively correlated with the levels of FFA release and serum FFAs, *n* = 5. **C** The image of primary preadipocytes and mature adipocytes and oil red O-stained mature adipocytes. **D** IH stimulation increased the levels of FFA release. **E** IH stimulation increased the levels of glycerol release. **F** The protein levels of ATGL, HSL and MGLL of mature adipocytes cultured with normoxia condition or IH condition. *n* = 9, *P < 0.05, **P < 0.01, ns indicates not significant, scale bar = 100 nm.

First, we collected adipose tissues from OSAS and control patients and found that lipids from OSAS patients had higher levels of FFA release than those from controls (Fig. 1A). In addition, the apnea hypopnea index (AHI) was determined by polysomnography and positively correlated with the levels of FFA release and serum FFAs (Fig. 1B), indicating a relationship between IH and FFA release. Next, we isolated primary preadipocytes and induced them to differentiate into mature adipocytes (Fig. 1C). We cultured mature adipocytes under normoxia conditions or under IH conditions, and lipolysis was measured. The results showed that the FFA and glycerol levels were increased in the supernatants in the IH group (Fig. 1D, E). In addition, we measured the protein levels of the crucial lipases ATGL, HSL and MGLL, and we found that the level of MGLL was significantly elevated by IH (Fig. 1F). Taken together, these results showed that IH induced adipocyte lipolysis.

### IH impaired the levels of the methylase METTL3 and m6A modifications

**Fig. 2:**
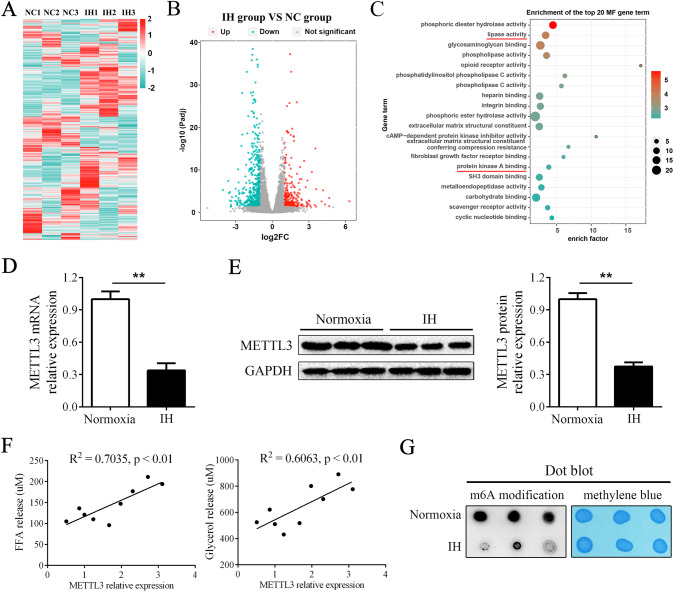
IH impaired the levels of the METTL3 and m6A modifications. **A**, **B** The heatmap and volcano plot of the RNA-Seq performed on adipocytes cultured with normoxia condition or IH condition. **C** The enrichment of the top 20 gene term from the GO analysis the DEGs. **D** IH stimulation downregulated the mRNA level of METTL3. **E** IH stimulation downregulated the protein level of METTL3. **F** The level of METTL3 was positively correlated with the levels of FFA release and glycerol release. **G** The dot blot showed that IH stimulation increased the total level of m6A modification in adipocytes. *n* = 9, **P < 0.01.

Next, we performed RNA-seq on adipocytes treated under normoxia or IH conditions. The results showed that 259 genes were upregulated and 458 genes were downregulated by IH (Fig. 2A, B). In addition, GO analysis showed that the differentially expressed genes (DEGs) were enriched in “lipase activity” and “protein kinase A binding” (Fig. 2C), which are closely related to lipolysis. Among the DEGs, METTL3 was notably reduced by IH treatment. Then, we examined the expression of METTL3 in a larger sample size, and the results showed that IH significantly downregulated the level of METTL3 in adipocytes (Fig. 2D, E). In addition, the expression of METTL3 showed a positive correlation with the levels of FFA release and glycerol release (Fig. 2F). Moreover, we measured the total level of m6A modification by dot blot analysis, and we found that IH reduced the m6A modification level in adipocytes (Fig. 2G). These findings indicated that METTL3-mediated m6A modifications participate in IH-related lipolysis.

### METTL3 inhibits lipolysis in adipocytes

**Fig. 3:**
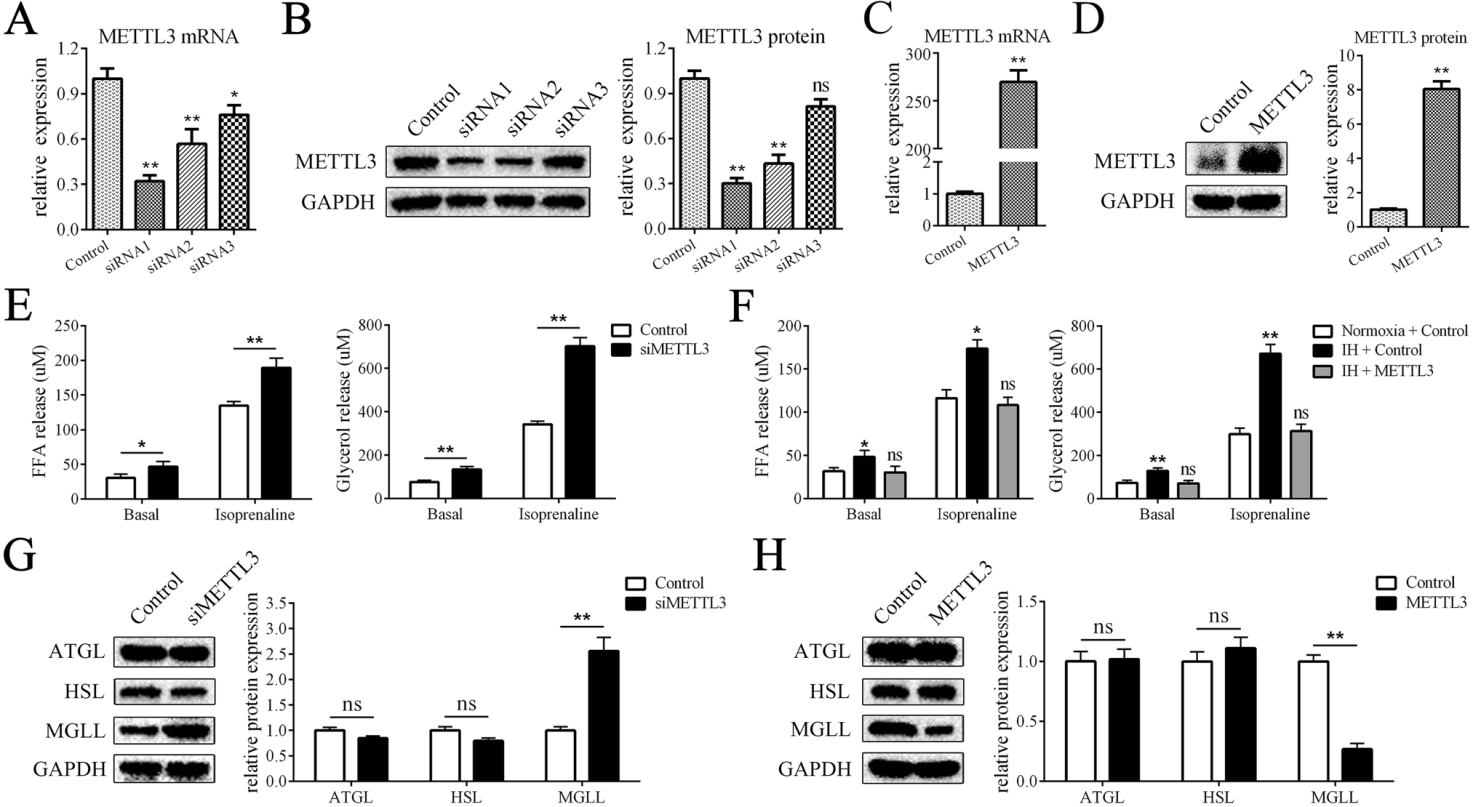
METTL3 inhibits lipolysis in adipocytes. **A**, **B** The mRNA and protein levels of METTL3 in adipocytes transfected with siRNAs. **C**, **D** The mRNA and protein levels of METTL3 in adipocytes transfected with control lentivirus or METTL3 lentivirus. **E** METTL3 downregulation increased the levels of FFA release and glycerol release. **F** IH stimulation increased the levels of FFA release and glycerol release and overexpression of METTL3 rescued these effects. **G** METTL3 downregulation increased the protein levels of MGLL but not ATGL or HSL. **H** METTL3 overexpression reduced the protein levels of MGLL but not ATGL or HSL; *n* = 9, *P < 0.05, **P < 0.01, ns indicates not significant. Control means treatment of negative control siRNA in **A**, **B**, **E**, **G** and means treatment of negative control lentivirus in **C**, **D**, **F**, **H**.

To determine the effect of METTL3 on lipolysis, we downregulated the expression of METTL3 in adipocytes with siRNAs. The silencing efficiencies of the siRNAs are shown in Fig. 3A, B, and the most effective siRNA was used for subsequent experiments. After METTL3 downregulation, the release of FFA and glycerol was significantly enhanced (Fig. 3E). In addition, the expression of MGLL but not AGTL or HSL was increased by siRNA targeting METTL3 (Fig. 3G). Furthermore, we overexpressed METTL3 with a lentivirus (Fig. 3C, D). The results showed that METTL3 overexpression abrogated the effect of IH on lipolysis (Fig. 3F). In addition, METTL3 overexpression significantly reduced the levels of MGLL but not AGTL or HSL (Fig. 3H). Taken together, these data showed that IH stimulated lipolysis through the downregulation of METTL3 expression.

### METTL3 inhibited lipolysis by downregulating MGLL expression

**Fig. 4:**
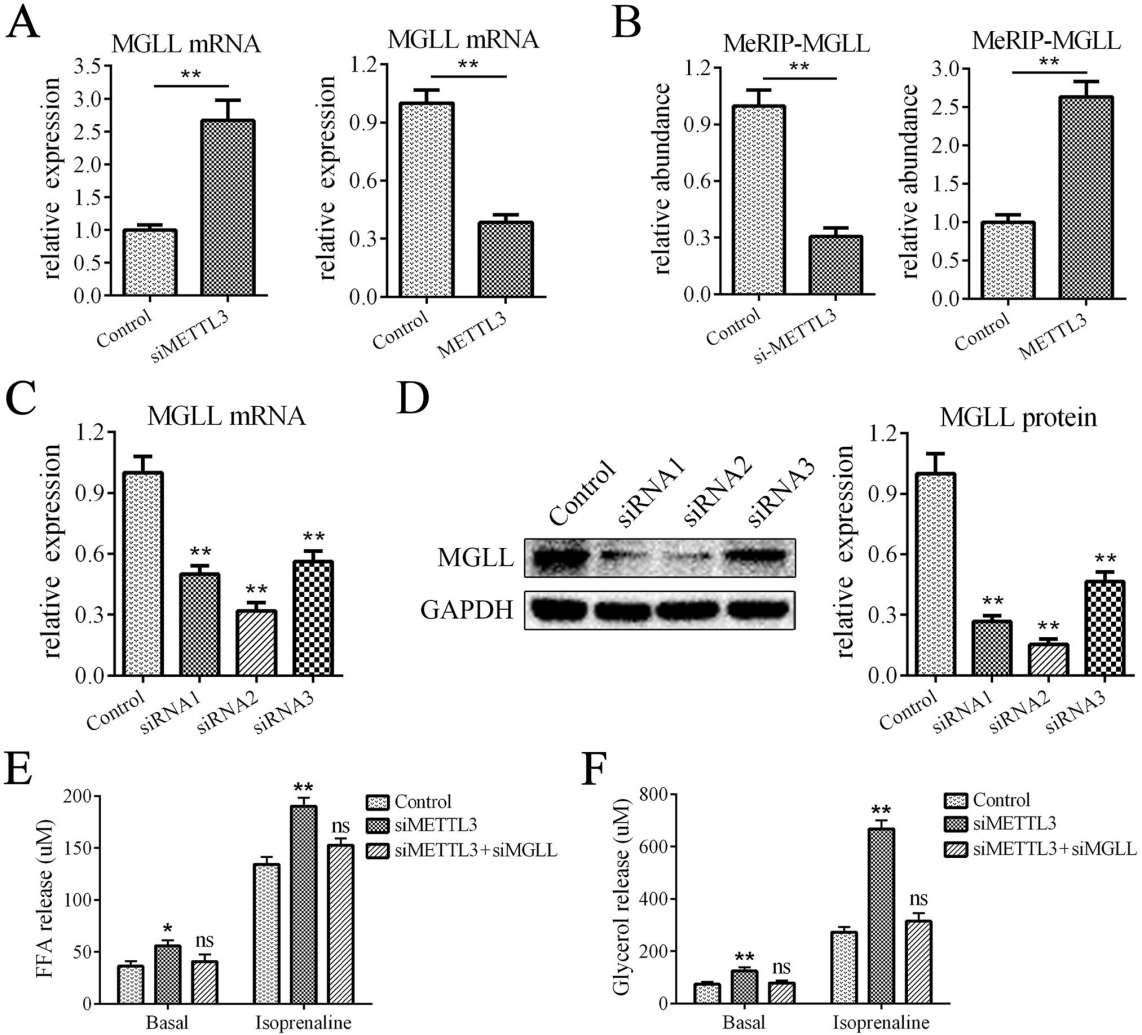
METTL3 inhibited lipolysis by downregulating MGLL expression. **A** The mRNA levels of MGLL were increased by METTL3 downregulation and decreased by METTL3 overexpression. **B** MeRIP-qPCR showed that the levels of m6A modification on MGLL mRNA were decreased by METTL3 downregulation and increased by METTL3 overexpression. **C**, **D** The mRNA and protein levels of MGLL in adipocytes transfected with siRNAs. **E**, **F** METTL3 downregulation increased the levels of FFA release and glycerol release and downregulation of MGLL rescued these effects; *n* = 9, *P < 0.05, **P < 0.01, ns indicates not significant. Control means treatment of negative control siRNA in **A** (left), **B** (left), **C**-**F** and means treatment of negative control lentivirus in **A** (right), **B** (right).

How does METTL3 mediate lipolysis? Because METTL3 serves as a crucial m6A methylase and the expression of the crucial lipase MGLL was significantly elevated by METTL3 downregulation, we examined whether METTL3 mediates the expression of MGLL via m6A modification and therefore regulates lipolysis. First, we showed that downregulating METTL3 enhanced the mRNA expression of MGLL, while METTL3 overexpression reduced the mRNA expression of MGLL (Fig. 4A). Second, the MeRIP-qPCR results showed that METTL3 knockdown impaired the m6A level on MGLL mRNA, while METTL3 overexpression had the opposite effect (Fig. 4B). Furthermore, we downregulated the expression of MGLL with siRNAs (Fig. 4C, D) and found that the effect of METTL3 downregulation on lipolysis was reversed (Fig. 4E, F). These results indicated that METTL3 promoted lipolysis through m6A-mediated upregulation of MGLL expression.

### METTL3 accelerated the degradation of MGLL mRNA via YTHDF2

**Fig. 5:**
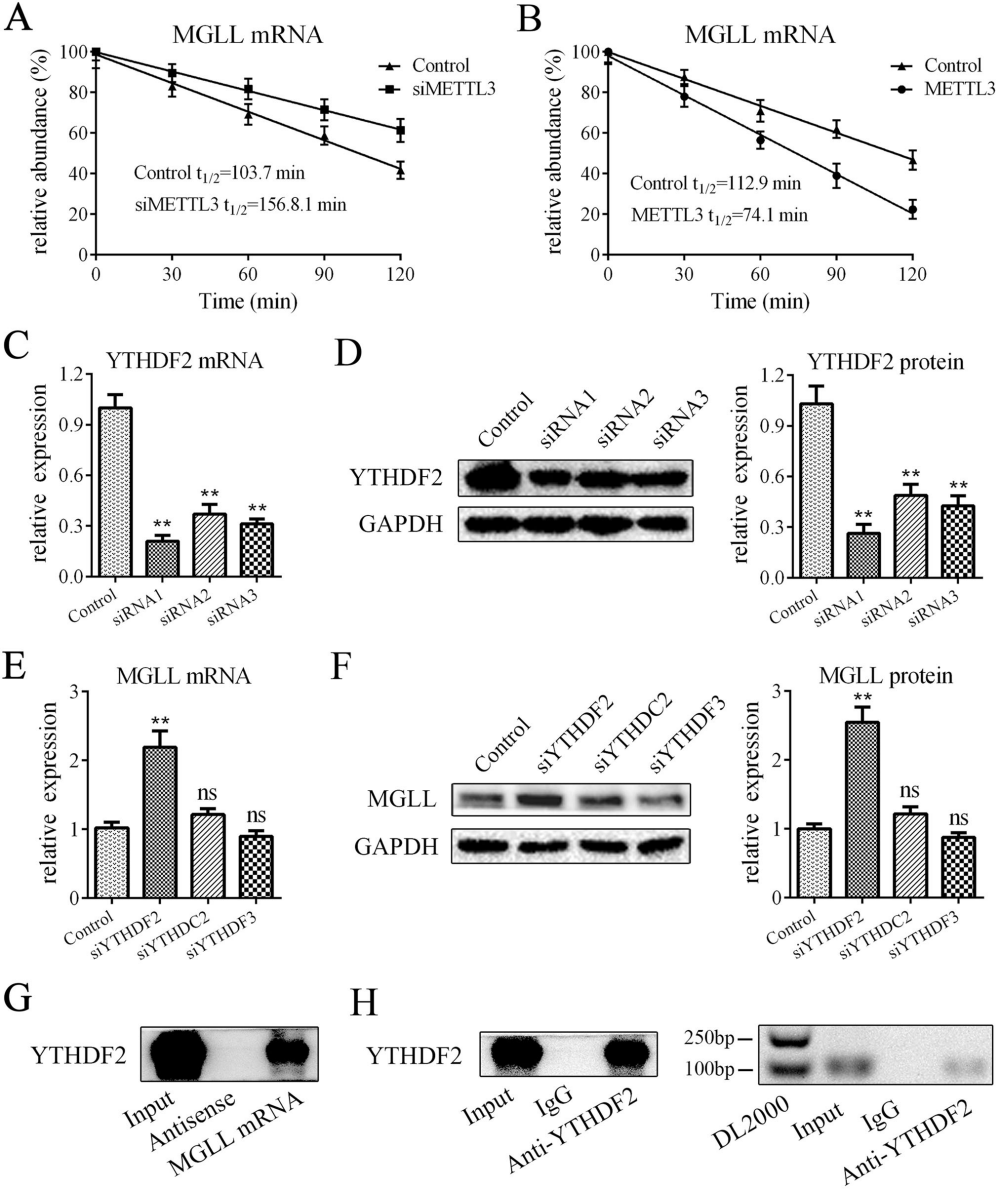
METTL3 accelerated the degradation of MGLL mRNA via YTHDF2. **A** METTL3 downregulation increased the half-life of MGLL mRNA. **B** METTL3 overexpression reduced the half-life of MGLL mRNA. **C**, **D** The mRNA and protein levels of YTHDF2 in adipocytes transfected with siRNAs. **E**, **F** The mRNA and protein levels of MGLL in adipocytes transfected with siRNAs targeting for YTHDF2, YTHDC2 or YTHDF3. **G** RNA pulldown showed that the YTHDF2 protein was interacted with MGLL mRNA but not antisense RNA. **H** RIP assay showed that MGLL mRNA was precipitated by YTHDF2 protein but not control IgG; *n* = 9, **P < 0.01, ns indicates not significant. Control means treatment of negative control siRNA in **A**, **C**, **D**-**F** and means treatment of negative control lentivirus in **B**.

We further explored how METTL3 modulated the expression of MGLL. Because the m6A modification mainly affects the stability of RNA and because the level of MGLL mRNA is regulated by METTL3, we examined whether METTL3 influences the degradation rate of MGLL mRNA. After transcriptional inhibition by actinomycin D, we found that the half-life of MGLL mRNA was significantly longer in the METTL3-knockdown group (Fig. 5A) and shorter in the METTL3-overexpression group (Fig. 5B), indicating that METTL3 impaired the stability of MGLL mRNA. Furthermore, we looked for the m6A reader that was responsible for MGLL mRNA. According to the literature, the three m6A readers YTHDC2, YTHDF2 and YTHDF3 have been reported to impair the stability of mRNA. We silenced these genes with siRNAs (Figs. 5C, D and S1A–D), and the results showed that the mRNA and protein levels of MGLL were enhanced by the siRNA targeting YTHDF2 but not the other two (Fig. 5E, F). In addition, we performed RNA pulldown assay, and the results showed that the YTHDF2 protein was integrated with MGLL mRNA (Fig. 5G), which was further verified by the RIP assay results (Fig. 5H). Taken together, these data showed that METTL3 accelerated the degradation of MGLL mRNA via the m6A reader YTHDF2.

### METTL3 reduced FFA levels and improved IR in CIH rats

**Fig. 6:**
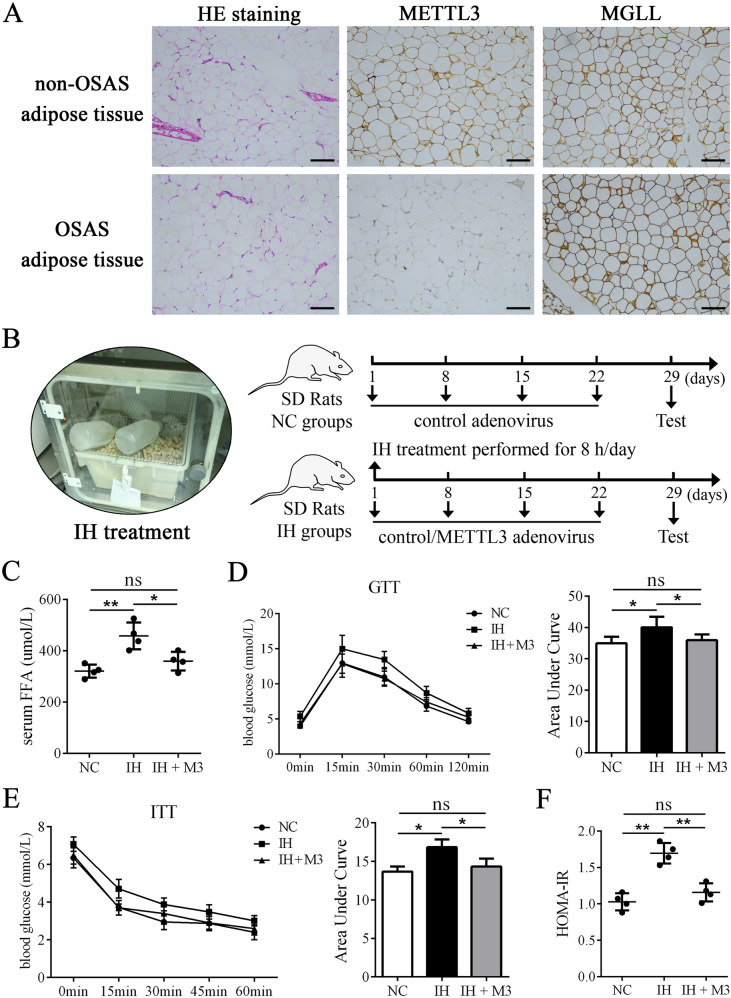
METTL3 reduced FFA levels and improved IR in CIH rats. **A** The HE staining and immunohistochemical staining of METTL and MGLL on adipose tissues from OSAS patients and non-OSAS donors. **B** The schematic diagram of CIH rats construction and adenovirus injection. **C** The levels of serum FFAs were enhanced in CIH rats and reduced by adenovirus overexpressing METTL3. **D** The levels of glucose tolerance detected by GTT were decreased in CIH rats and improved by adenovirus overexpressing METTL3. **E** The levels of insulin sensitivity detected by ITT were decreased in CIH rats and improved by adenovirus overexpressing METTL3. **F** The HOMA-IR was increased in CIH rats and reduced by adenovirus overexpressing METTL3; *n* = 4, *P < 0.05, **P < 0.01, ns indicates not significant. M3 means treatment of adenovirus overexpressing METTL3.

In vivo, we first performed IHC on adipose tissues from OSAS patients and control donors, and the results showed that the METTL3 signal was weakened while the MGLL signal was strengthened in OSAS patients (Fig. 6A). In addition, we constructed a CIH rat model, and an adenovirus overexpressing METTL3 was injected (Fig. 6B). CIH rats exhibited increased levels of serum FFAs (Fig. 6C) and decreased glucose tolerance and insulin sensitivity (Fig. 6D, E). In addition, HOMA-IR was elevated in CIH rats (Fig. 6F). Treatment with METTL3 adenovirus not only reduced serum FFA levels but also improved the glucose tolerance, insulin sensitivity and HOMA-IR in CIH rats (Fig. 6C to F). These data suggested that METTL3 reduced FFA levels and protected against IR caused by CIH in vivo.

## Discussion

In this study, we focused on the effect of IH on lipolysis in OSAS and found that IH facilitated lipolysis via m6A modifications. IH decreased the expression of METTL3, which reduced the level of the m6A modification on MGLL mRNA and enhanced MGLL mRNA stability via YTHDF2. In addition, the level of METTL3 was decreased in lipids from OSAS patients, and METTL3 lowered FFA levels and improved IR in CIH rats. Our findings provide new insights into the pathogenesis of IR in OSAS and provide potential therapeutic targets.

Previous studies have shown that OSAS is an independent risk factor for IR and that IH is the cause and therapeutic target for IR in OSAS. Punjabi and other studies have shown that IR is associated with the AHI in OSAS patients [17]. Polak et al. [4] showed that in mice, IH led to IR and abnormal glucose metabolism. Louis and Punjabi [5] showed that exposure of healthy volunteers to IH could decrease insulin sensitivity. However, little is known about the mechanism by which IH affects IR. Previous studies have shown that the levels of FFA, which are key factors in the occurrence and development of IR, were enhanced in OSAS patients [11]. Here, our data showed that the level of IH in OSAS, as indicated by the AHI, was positively correlated with serum FFA levels and FFA release from adipocytes. In addition, we revealed that IH facilitated lipolysis and FFA release in adipocytes, providing new insights into the development of IR in OSAS. Currently, CPAP and surgery are two main methods to improve IH in OSAS. However, poor compliance and surgical risks are associated with these two methods [18]. In this study, we showed that the injection of an adenovirus overexpressing METTL3 reduced FFA levels and improved IR in CIH rats. This finding suggested that targeting METTL3 was a potential method to fight IH-mediated IR in OSAS, and this conclusion requires further investigation.

Lipolysis is a straightforward process that releases FFAs from adipose tissue, and this process is precisely regulated by hormonal and biochemical signals in response to changes in nutritional state or environmental stimuli [8]. IH is a common stress that is present in some pathological conditions, such as OSAS or chronic obstructive pulmonary disease [19]. Previous studies have examined the effect of IH on serum FFA levels and adipokines in adipocytes and suggested a correlation between IH and lipolysis [20, 21]. However, whether and how IH participates in lipolysis and FFA release in adipocytes has not been reported. In this study, we first explored the effect of IH on FFA release at the cellular and molecular levels and found that IH stimulated MGLL-mediated lipolysis and FFA release. ATGL, HSL and MGLL are three core lipolytic enzymes [8]. Previous studies mainly focused on ATGL and HSL and showed that these factors were involved in various human diseases, such as diet-induced IR, type 2 diabetes and lipodystrophy [22, 23]. However, the role of MGLL in human disease is ambiguous. Here, we revealed a role of MGLL in IH-mediated FFA release and IR in OSAS. Consistently, a study showed that MGLL deficiency impaired lipolysis and attenuated IR in mice [24]. We consider MGLL to be a potential target for the prevention and treatment of IR.

The m6A modification has become a hot topic in recent years, and METTL3 was reported to participate in fat metabolism and IR [25, 26]. Qiu’s research revealed that interfering with METTL14 rescued arsenic-impaired hepatic insulin sensitivity [27]. Nenja showed that the loss of endothelial FTO protected mice from high-fat diet-induced glucose intolerance and IR [28]. However, the effect of METTL3 is contradictory. Our data showed that METTL3 improved CIH-induced IR. Consistent with our results, Wang revealed that specific deletion of METTL3 in adipose tissue severely promoted high-fat diet (HFD)-induced obesity and IR [26]. While Li showed that specific knockout of METL3 in hepatocytes significantly reduced lipid accumulation and improved insulin sensitivity [29]. We believe that the effect of METTL3 on IR varies in different cell types. Further investigation is required to elucidate the effect of METTL3 on various cell types and IR, which will guide us in the precise treatment of IR.

The effect of the m6A modification on transcription requires the activity of m6A readers, and different readers exert various effects [15]. For example, YTHDC1 was reported to regulate the splicing and transport of transcripts [15]. IGF2BPs was reported to enhance the stability of transcripts, while YTHDF2 accelerated the degradation of m6A-modified RNA [30, 31]. Here, we identified that YTHDF2 interacted with MGLL mRNA and impaired its stability, which is consistent with previous reports. Previous studies have examined the effect of m6A readers on lipolysis and IR. Alvarez showed that IGF2BP2 downregulation impaired adipocyte lipolysis [32]. Zhou revealed that Ythdc2 could bind to the mRNA of lipogenic genes to improve liver steatosis and IR in mice [33]. Here, we also revealed that YTHDF2 participated in adipocyte lipolysis and IR. Taken together, these results indicate that m6A readers play important roles in the regulation of lipolysis and IR and may possess clinical application value.

In conclusion, we revealed a role for METTL3 in the regulation of IH-induced adipocyte lipolysis and FFA release and provided new insights into the prevention and treatment of IR in OSAS.

## Materials and methods

### Study subjects

The study subjects consisted of 5 patients with OSAS undergoing polysomnography without CPAP treatment and 5 control patients without OSAS or IR. The exclusion criteria for all subjects included the presence of congenital heart disease, myocardial infarction, congestive heart failure, circadian rhythm disorder, chronic insufficient sleep, obstructive lung diseases or lung surgery. All participants underwent abdominal operations for gastrointestinal disorders, and adipose tissues were collected during the operation without additional injury. Informed consent was obtained from every participant. The clinical characteristics of all participants are provided in Table S1.

### Isolation and adipogenic induction of primary preadipocytes

Primary preadipocytes were isolated from subcutaneous adipose tissue collected from 9 donors who underwent abdominoplasty as previously described [34]. Briefly, adipose tissue was cut into pieces and digested with 0.2% type I collagenase for 45 min. Then, two volumes of DMEM/F12 medium supplemented with 15 mmol/L HEPES, 14 mmol/L NaHCO_3_, 33 μmol/L biotin, 17 μmol/L D-pantothenate, and 10% FCS were added. The mixture was filtered, and the cell suspension was collected and centrifuged. The precipitated cells were resuspended and seeded in culture flasks. The cells were cultured in DMEM/F12 medium with 5% FCS in an incubator at 37 °C with 5% CO_2_. When the cells reached 80–90% confluence, they were digested with trypsin and divided into two flasks. The induction of adipogenic differentiation was performed on primary preadipocytes at passage 3. The adipogenic medium was composed of DMEM supplemented with 10% FBS, 0.5 mM IBMX, 1 mM dexamethasone, 10 mg/ml insulin, 0.2 mM indomethacin, 100 IU/ml penicillin, and 100 IU/ml streptomycin (all from Sigma). The medium was replaced every 3 days until day 14, and the cells were examined with oil red O staining or used for other experiments.

### IH treatment

IH treatment was performed as previously described [35]. Briefly, mature adipocytes were cultured in a hypoxia incubator for 35 min, followed by incubation in a normoxia incubator. The hypoxia incubator was filled with mixed air composed of 1% O_2_ and 5% CO_2,_ and the normoxia incubator was filled with 21% O_2_ and 5% CO_2_. IH treatment was conducted for 6 cycles per day.

### FFA and glycerol measurement

Serum was isolated from peripheral blood by centrifugation and stored at −80 °C until analysis. Cell supernatant was collected after the cells were cultured with basal condition or with isoprenaline stimulation for 24 h and immediately analyzed. Adipose tissue was freshly collected and processed immediately as previously described [36]. A Free Fatty Acid Assay Kit (Nanjing Jiancheng, A042-2) and a Glycerol Assay Kit (Nanjing Jiancheng, F005-1) were used to measure FFA and glycerol levels according to the manufacturer’s instructions.

### Quantitative polymerase chain reaction (qPCR)

The RNA was extracted using TRIzol™ Reagent (Thermo Fisher, 15596-026) according to the manufacturer’s instructions. The concentrations of the extracted RNA were measured and equal amount of RNA was reversely transcribed into cDNA using Evo M-MLV RT Master Mix (Accurate Biology, AG11706). The qPCR was performed on cDNA by using SYBR^®^ Green Pro Taq HS Premix (Accurate Biology, AG11701) on a Real-time fluorescence quantitative PCR system (Applied Biosystems, ABI7500). The relative RNA levels were analyzed in 2^−ΔΔCt^ method with GAPDH as the reference gene. The primers used in this study are showed in Table S2.

### Protein extraction and western blot

The cell protein was extracted by using RIPA buffer followed with centrifuge at 14,000 rpm for 30 min at 4 °C. Then, the protein concentrations were detected using a BCA Protein Assay Kit (CWBIO, CW0014S) and equal amount of protein were mixed with sodium dodecyl sulfate (SDS) loading buffer. The mixtures were boiled for 10 min and stored at −80°Cor used directly. For western blot assay, the SDS-page gels (Beyotime, P0012A) were prepared according to the instruction and the proteins were separated via electrophoresis. Then the proteins were transferred to polyvinylidene fluoride membranes (Millipore, IPVH0010) and incubated with primary antibodies at 4 °C overnight after being blocked by 5% non-fat milk. On the second day, the membranes were washed and incubated with HRP-conjugated secondary antibodies for 1 h at room temperature. The protein signals were detected by Immobilon Western HRP Substrate (Millipore, WBKLS0500) and the gray values were analyzed by image J.

### RNA sequencing

RNA sequencing (RNA-seq) was performed as previously described [37]. Briefly, Adipocytes from three different donors were cultured with normoxia condition or IH condition and the RNA were extracted by Trizol solution. Then the RNA was fragmented and reverse-transcribed into cDNA. After that, terminal repair and sequencing connection was performed following with PCR amplification. Finally, the sequence was performed by using Illumina HiSeq at Qiantang Biotechnology (Suzhou) Co., Ltd.

### Dot blot analysis

The dot blot assay was performed as previously described [38]. Briefly, 100 ng of RNA from each sample was added to two nylon membranes (Sigma–Aldrich, GERPN1210B), and UV crosslinking was performed. One of the membranes was stained with methylene blue solution and imaged with a camera. The other membrane was blocked with 5% nonfat milk for 1 h at room temperature. Subsequently, the membrane was incubated with m6A methylation antibody overnight at 4 °C. Then, the membrane was washed and incubated with secondary antibodies for 1 h at room temperature. Finally, the membrane was washed and visualized by Immobilon Western HRP Substrate (Millipore, WBKLS0500).

### Methylated RNA immunoprecipitation-qPCR (MeRIP-qPCR)

The assay was performed by using an EZ-Magna RIP Kit (Merck Millipore, 17-701) according to the instructions. Briefly, the extracted RNA was fragmented and incubated with A/G magnetic beads that were premixed the m6A methylated antibody. The mixture was shaken gently at 4 °C overnight. Then, the conjugated RNA was purified and reverse transcribed into cDNA. Finally, qPCR was performed to measure the relative gene expression, and the abundance of m6A on the RNA is expressed as input%.

### RNA pulldown

The plasmids overexpressing mature MGLL mRNA or corresponding antisense transcripts were constructed by OBIO Technology (Shanghai, China). The mature MGLL mRNA or corresponding antisense transcripts were transcribed in vitro using a TranscriptAid T7 High-Yield Transcription Kit (Thermo Fisher, K0441). The transcripts were extracted and used for RNA pulldown by using a Pierce™ Magnetic RNA-Protein Pull-Down Kit (Thermo Fisher, 20164) according to the manufacturer’s instructions. The pulled-down proteins were purified and the expression of YTHDF2 was detected by western blot assay.

### RNA immunoprecipitation (RIP)

An EZ-Magna RIP™ RNA-Binding Protein Immunoprecipitation Kit (Millipore, 17-701) was used as the manufacturer’s instructions. Briefly, MSCs were lysed and incubated with magnetic beads conjugated with anti-YTHDF2 (Abcam, ab220163, 1:30) or negative control IgG (Santa Cruz, sc-3877, 1:100). The qPCR and electrophoresis were used to measure the abundance of MGLL mRNA.

### RNA degradation rate detection

The actinomycin D (2ug/ml) was added to the culture medium and the cells were lysed TRIzol™ Reagent after 0 min, 30 min, 60 min, 90 min and 120 min. The relative abundance of the MGLL was detected by qPCR and the RNA degradation rate was analyzed.

### CIH model construction and analysis

The CIH model was constructed as previously described [39]. Twelve adult male SD rats were randomly divided into three groups: the normoxia control group (NC), the IH group (IH) and the IH + METTL3 group (IM). IH treatment was performed with an intermittent oxygen environment control system (Yuyan Instrument, S1007, China) provided by Forevergen Company (China). Rats were fed a standard diet and kept on a 12-h light/dark schedule (08.00 h–20.00 h). IH treatment was performed for 8 h/day from 8:00 AM to 4:00 PM for 28 days, and the oxygen concentration oscillated between 21 and 5% with a period of 60 s. Rats in the NC and IH groups were injected with the control adenovirus, and rats in the IM group were injected with METTL3 adenovirus on days 1, 8, 15 and 22. Fasting blood glucose (FBG) and serum FFA, urea and creatinine levels were measured, and glucose tolerance tests (GTTs) and insulin tolerance tests (ITTs) were performed on day 29.

### Immunohistochemistry (IHC)

Freshly collected adipose tissues were fixed with 10% neutral formalin for 24 h and embedded in paraffin. Then, the tissues were sliced, and IHC was performed by using an SP Rabbit & Mouse HRP Kit (CWBIO, CW2069) according to the instructions. Briefly, the slides were deparaffinized in fresh xylene and rehydrated in an ethanol gradient. Then, antigen retrieval was performed with pepsase, and the sections were blocked with 10% goat serum for 30 min. Next, the sections were incubated with anti-METTL3 (Abcam, ab195352, 1:500) and anti-MGLL (Proteintech, 14986-1-A, 1:300) overnight at 4 °C, followed by incubation with secondary antibodies and DAB substrate. The slides were washed and counterstained with hematoxylin, and images were captured under a light microscope.

### Statistical analyses

The data of this study were expressed as the mean ± standard deviation (SD) and analyzed by SPSS 22.0 software. The analysis of differences between groups was performed by using independent-sample t-tests. The analysis of correlation was performed by Pearson correlation and linear regression analysis. *P* < 0.05 was regarded as significant. The sample sizes and the statistical results were included in the figure legends.

## Supplementary information

Figure S1

Supplemental Figure Legends

Supplemental Tables

Supplemental materials and methods

Full length western blots

## Data Availability

The datasets used and/or analyzed during the current study are available from the corresponding authors on reasonable request.
