# Supplementary figures and images for: Intermittent hypoxia-induced METTL3 downregulation facilitates MGLL-mediated lipolysis of adipocytes in OSAS
Source: Cell Death Discov. 2022 Aug 6;8:352. doi: 10.1038/s41420-022-01149-4 (PMC9357002; doi:10.1038/s41420-022-01149-4)

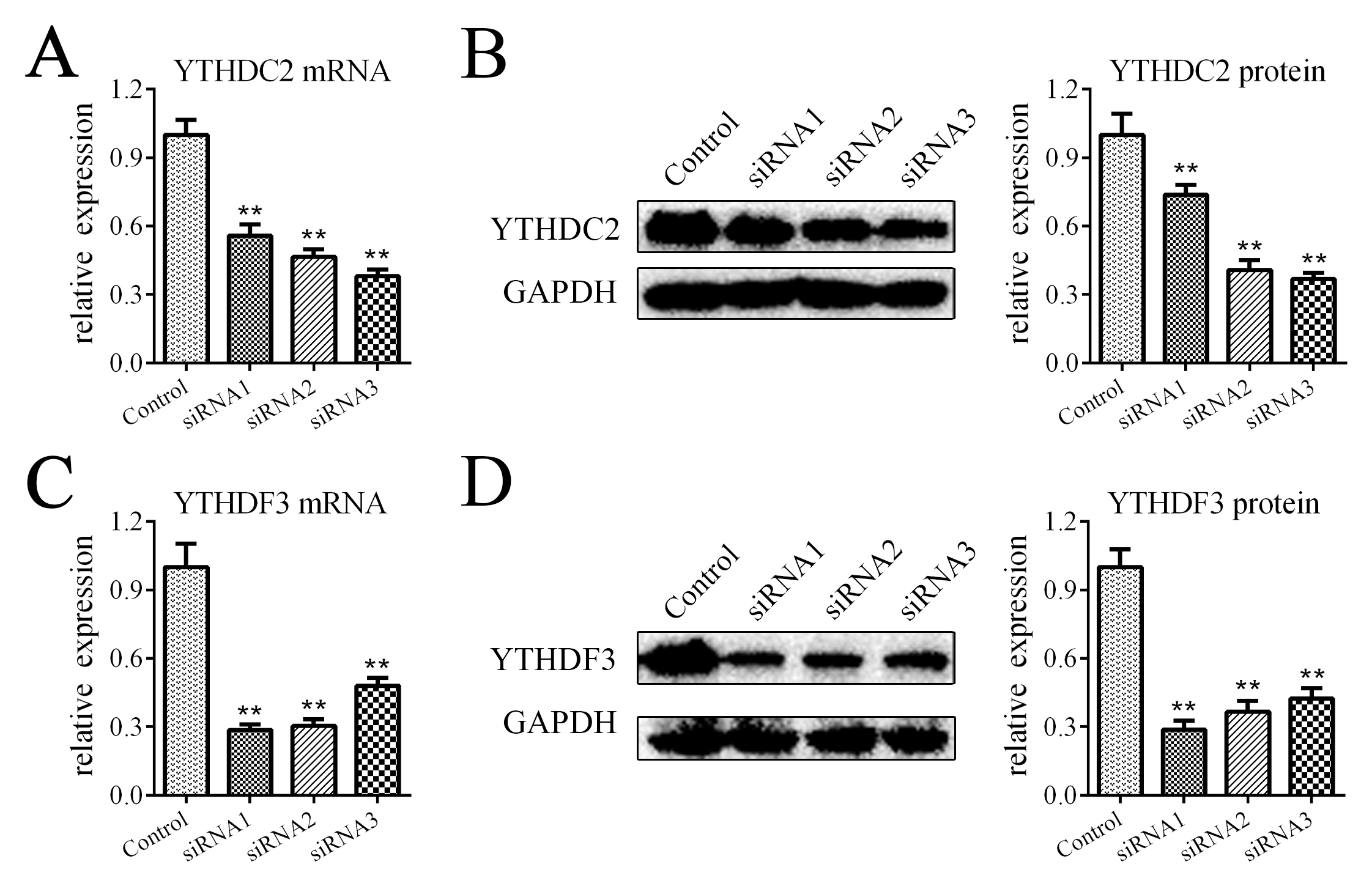

Supplement: Supplementary file 1 — Figure S1 [file 41420_2022_1149_MOESM1_ESM.jpg]
