# Supplementary material for: Intermittent hypoxia-induced METTL3 downregulation facilitates MGLL-mediated lipolysis of adipocytes in OSAS
Source: Cell Death Discov. 2022 Aug 6;8:352. doi: 10.1038/s41420-022-01149-4 (PMC9357002; doi:10.1038/s41420-022-01149-4)
Supplement: Supplementary file 2 — Supplemental Figure Legends [file 41420_2022_1149_MOESM2_ESM.docx]

**Supplemental Figure Legends**

**Figure S1**. The silencing efficiencies of the siRNAs targeting YTHDC2 or YTHDF3. **A - B.** The mRNA and protein levels of YTHDC2 in adipocytes transfected with siRNAs targeting YTHDC2; **C - D.** The mRNA and protein levels of YTHDF3 in adipocytes transfected with siRNAs targeting YTHDF3; n = 9, ** indicates P < 0.01. Control means treatment of negative control siRNA.
