# Supplementary material for: Intermittent hypoxia-induced METTL3 downregulation facilitates MGLL-mediated lipolysis of adipocytes in OSAS
Source: Cell Death Discov. 2022 Aug 6;8:352. doi: 10.1038/s41420-022-01149-4 (PMC9357002; doi:10.1038/s41420-022-01149-4)
Supplement: Supplementary file 3 — Supplemental Tables [file 41420_2022_1149_MOESM3_ESM.docx]

**Supplemental Tables**

| **Characteristics** | **OSAS patients** | **Non-OSAS donors** | **p-value** |
| --- | --- | --- | --- |
| Numbers | 5 | 5 | ns |
| Age, year | 46.8 ± 3.30 | 52.4 ± 3.33 | ns |
| Sexuality | Male: 3, Female: 2 | Male: 3, Female: 2 | ns |
| BMI (body mass index) | 26.2 ± 1.31 | 23.82 ± 1.43 | ns |
| AHI (times/hour) | 21.6 ± 2.72 | 2.37 ± 0.46 | < 0.01 |
| Serum FFA (umol/L) | 526.3 ± 16.74 | 425.5 ± 14.49 | < 0.01 |

**Table S1. Characteristics of the study subjects**

**Abbreviations**: OSAS, obstructive sleep apnea syndrome; AHI, apnea hypopnea index; FFA, free fatty acid; ns, not significant. All data were presented as mean ± standard error of mean (SEM).

**Table S2. Primer sequences**

| **Gene names** | **Forward primer (5' - 3')** | **Reverse primer (5' - 3')** |
| --- | --- | --- |
| GAPDH | GGAGCGAGATCCCTCCAAAAT | GGCTGTTGTCATACTTCTCATGG |
| METTL3 | TTGTCTCCAACCTTCCGTAGT | CCAGATCAGAGAGGTGGTGTAG |
| MGLL | TCGTCAGGGATGTGTTGCAG | AGGCGAAATGAGTACCATGCC |
| YTHDC2 | CAAAACATGCTGTTAGGAGCCT | CCACTTGTCTTGCTCATTTCCC |
| YTHDF2 | CCTTAGGTGGAGCCATGATTG | TCTGTGCTACCCAACTTCAGT |
| YTHDF3 | GGTGTATTTAGTCAACCTGGGG | AAGAGAACTAGGTGGATAGCCAT |

**Table S3. siRNA sequences**

| **siRNA names** | **sense (5' - 3')** | **antisense (5' - 3')** |
| --- | --- | --- |
| NC | UUCUCCGAACGUGUCACGUTT | ACGUGACACGUUCGGAGAATT |
| METTL3-si-1 | GCUACCUGGACGUCAGUAUTT | AUACUGACGUCCAGGUAGCTT |
| METTL3-si-2 | GGUUGGUGUCAAAGGAAAUTT | AUUUCCUUUGACACCAACCTT |
| METTL3-si-3 | GGUGACUGCUCUUUCCUUATT | UAAGGAAAGAGCAGUCACCTT |
| MGLL-si-1 | GGAUGGUAGUGUCUGACUUTT | AAGUCAGACACUACCAUCCTT |
| MGLL-si-2 | GCCGGCAUGGUACUCAUUUTT | AAAUGAGUACCAUGCCGGCTT |
| MGLL-si-3 | UAUUAGACAUUUCAUCUUCTT | GAAGAUGAAAUGUCUAAUATT |
| YTHDF2-si-1 | UCCUUUUGAUGUACAGAUCTT | GAUCUGUACAUCAAAAGGATT |
| YTHDF2-si-2 | ACGAUGUUACUAGUAAUGGTT | CCAUUACUAGUAACAUCGUTT |
| YTHDF2-si-3 | UAAUGGAACGGUGAAUAUCTT | GAUAUUCACCGUUCCAUUATT |
| YTHDC2-si-1 | AGGAAAUUCCAUUUCUCUCTT | GAGAGAAAUGGAAUUUCCUTT |
| YTHDC2-si-2 | UCCAAAAUAUCUUAUAAAGTT | CUUUAUAAGAUAUUUUGGATT |
| YTHDC2-si-3 | UUUCUUUGUUUGUAUAUCCTT | GGAUAUACAAACAAAGAAATT |
| YTHDF3-si-1 | UUAAGUAUGGCUCAAAAUCTT | GAUUUUGAGCCAUACUUAATT |
| YTHDF3-si-2 | UUACUCAAAGUAUCAUUGCTT | GCAAUGAUACUUUGAGUAATT |
| YTHDF3-si-3 | UAAUGGAACGAUGUAUGUCTT | GACAUACAUCGUUCCAUUATT |
