# Supplementary material for: Intermittent hypoxia-induced METTL3 downregulation facilitates MGLL-mediated lipolysis of adipocytes in OSAS
Source: Cell Death Discov. 2022 Aug 6;8:352. doi: 10.1038/s41420-022-01149-4 (PMC9357002; doi:10.1038/s41420-022-01149-4)
Supplement: Supplementary file 4 — Supplemental materials and methods [file 41420_2022_1149_MOESM4_ESM.docx]

**Supplemental materials and methods**

**The antibodies used for western blot in this study:**

Anti-ATGL (Abcam, ab109251, 1:1000), Anti-MGLL (Proteintech, 14986-1-A,1:1000), Anti-HSL (Cell Signaling Technology, 4107T, 1:1000), Anti-METTL3 (Abcam, ab195352, 1:1000), Anti-YTHDC2 (Abcam, ab220160, 1:1000), Anti-YTHDF2 (Abcam, ab220163, 1:1000), Anti-YTHDF3 (Abcam, ab220161, 1:1000). Anti-GAPDH (Abcam, ab8245, 1:3000), HRP Conjugated AffiniPure Goat Anti-mouse IgG (Boster, BA1050, 1:3000), HRP Conjugated AffiniPure Goat Anti-rabbit IgG (Boster, BA1054, 1:3000).
