# Supplementary material for: Intermittent hypoxia-induced METTL3 downregulation facilitates MGLL-mediated lipolysis of adipocytes in OSAS
Source: Cell Death Discov. 2022 Aug 6;8:352. doi: 10.1038/s41420-022-01149-4 (PMC9357002; doi:10.1038/s41420-022-01149-4)

Full length western blots

Figure 1F-ATGL:


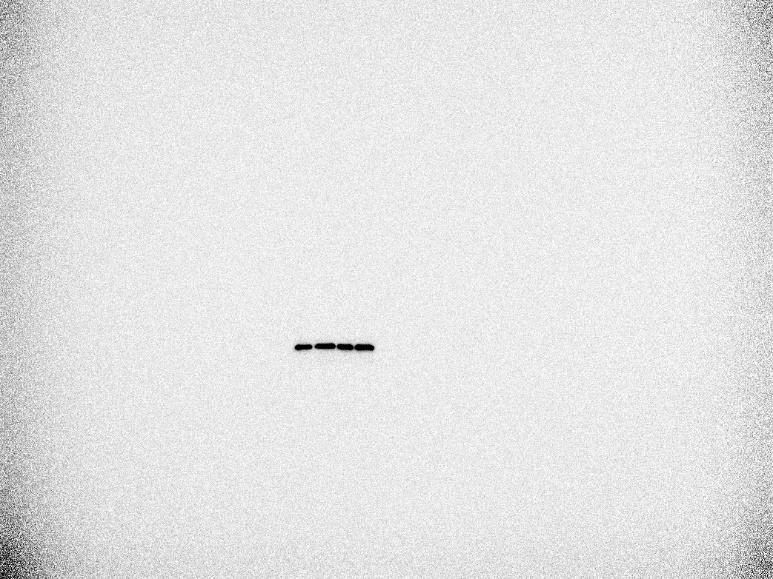


Figure 1F-HSL:


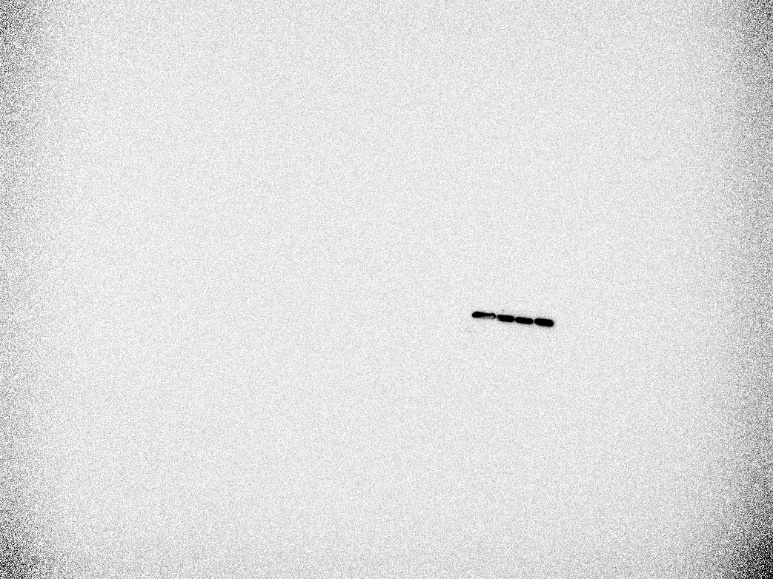


Figure 1F-MGLL:


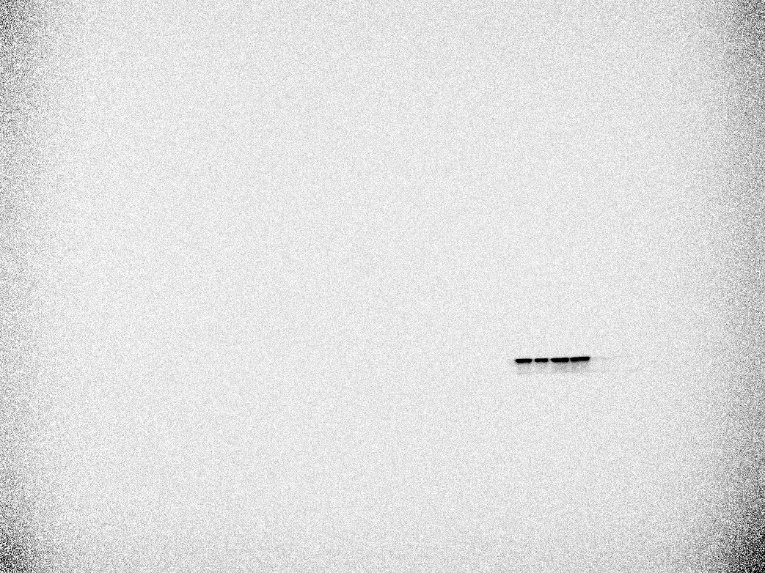


Figure 1F-GA:


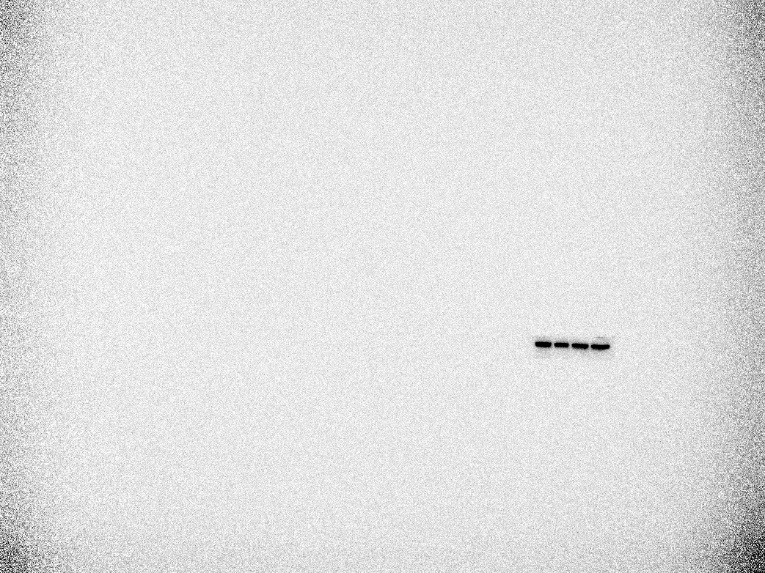


Figure 2E-METTL3:


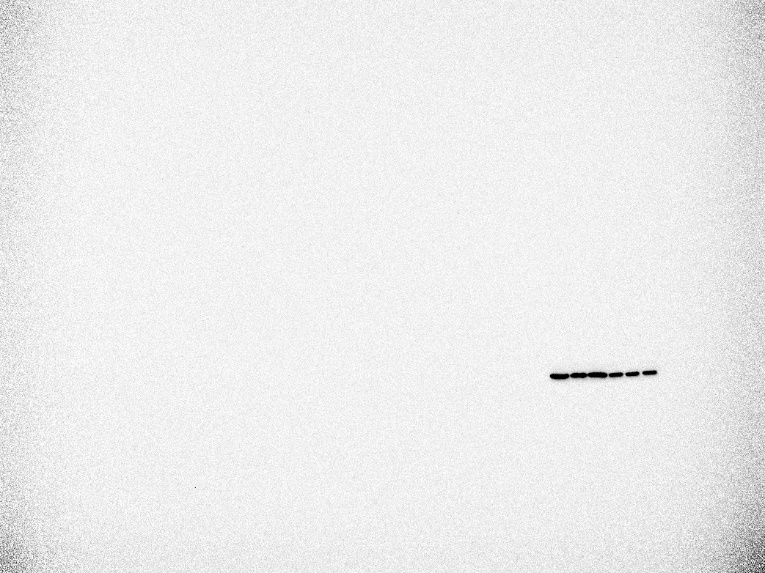


Figure 2E-GA:


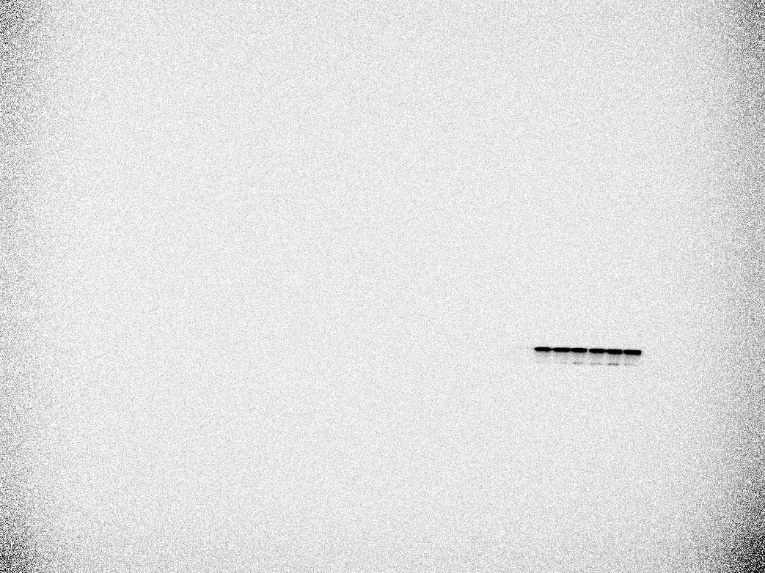


Figure 3B-METTL3:


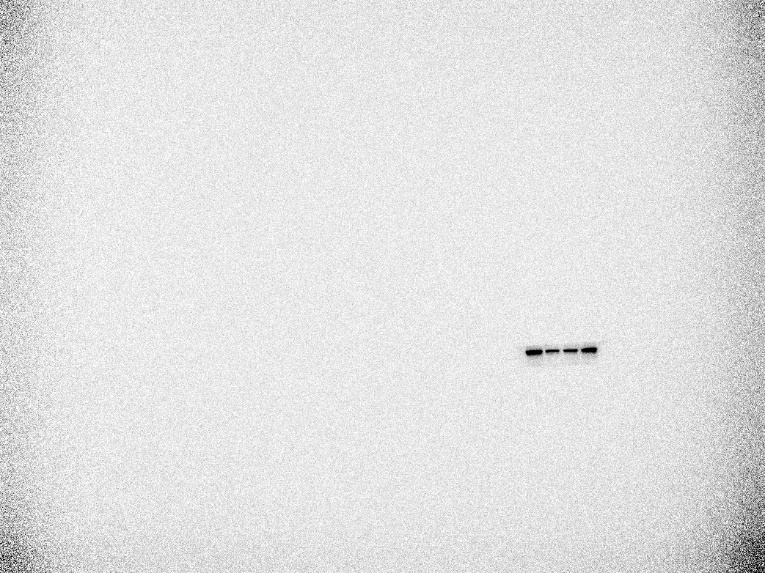


Figure 3B-GA:


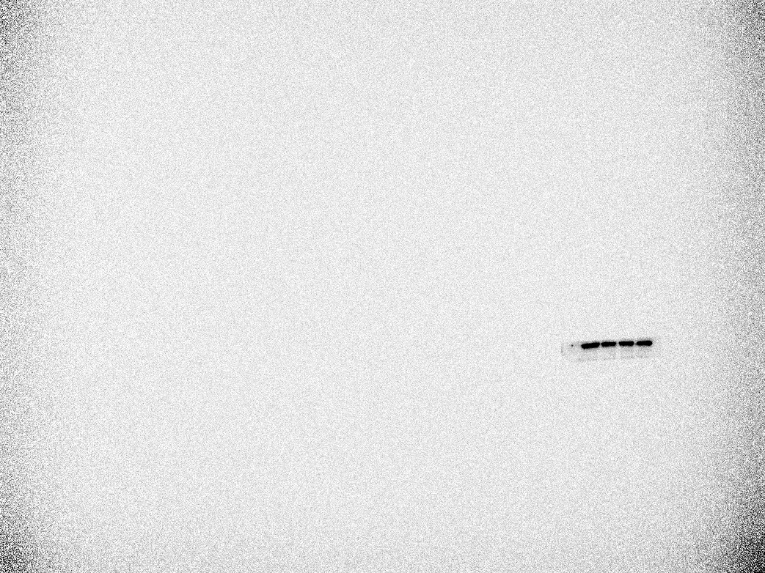


Figure 3D-METTL3:


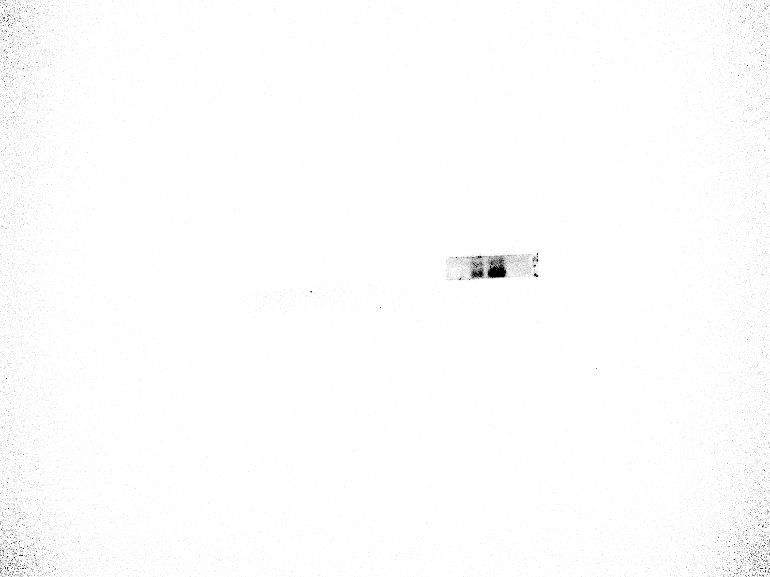


Figure 3D-GA:


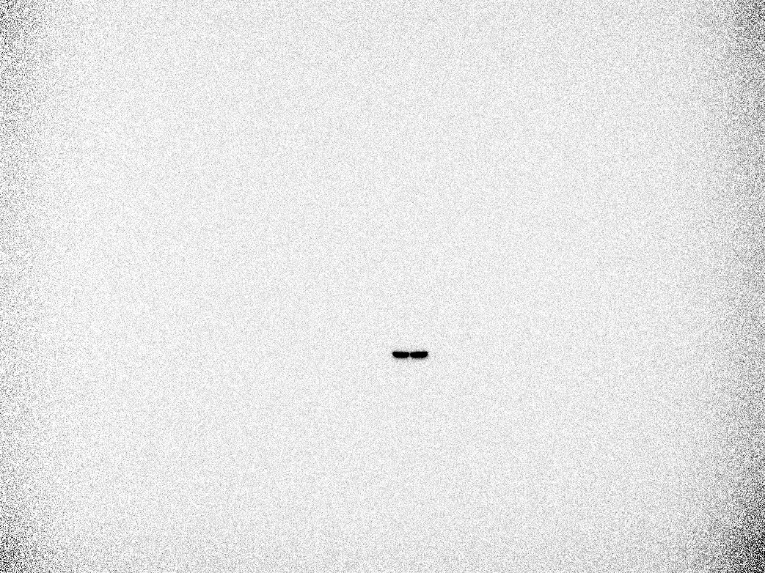


Figure 3H-ATGL:


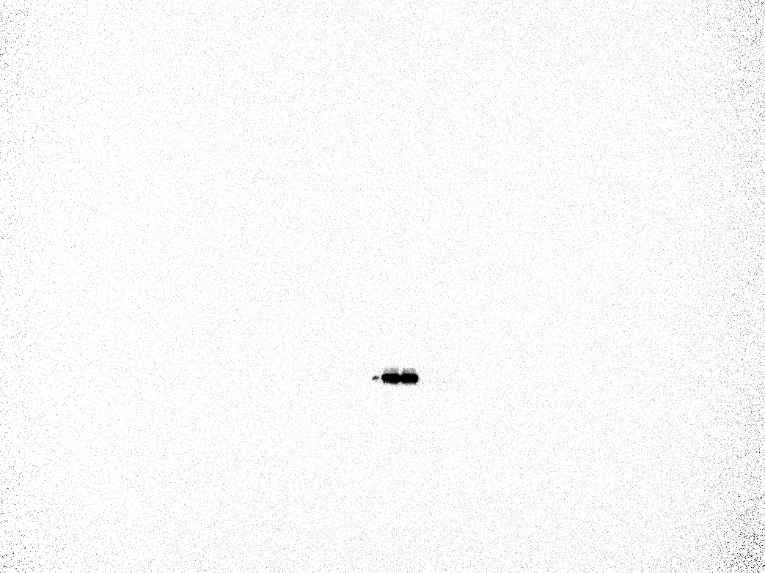


Figure 3H-HSL:


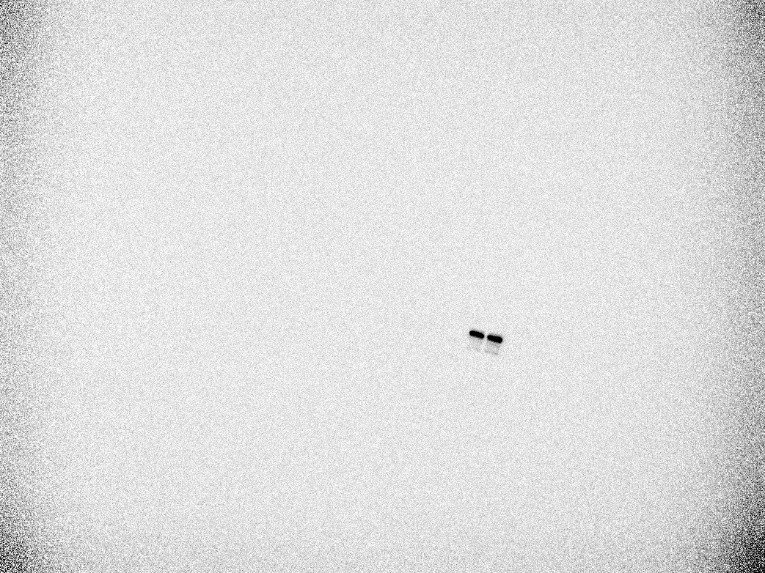


Figure 3H-MGLL:


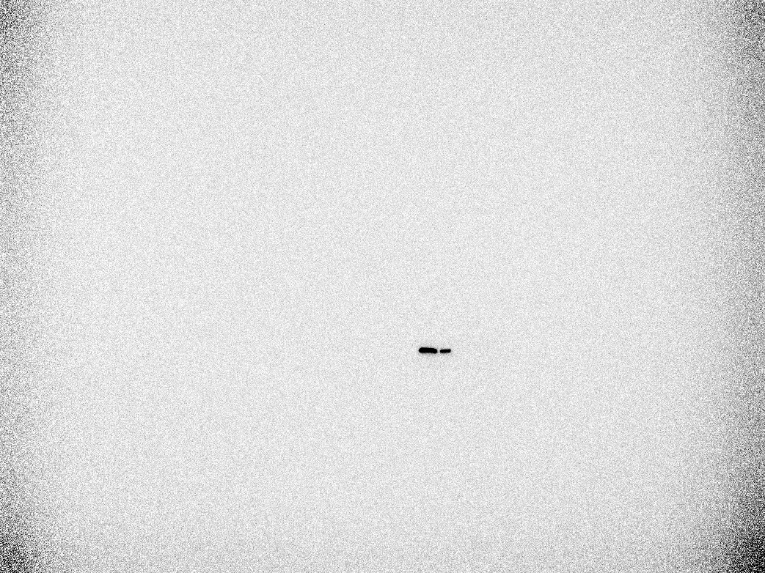


Figure 3H-GA:


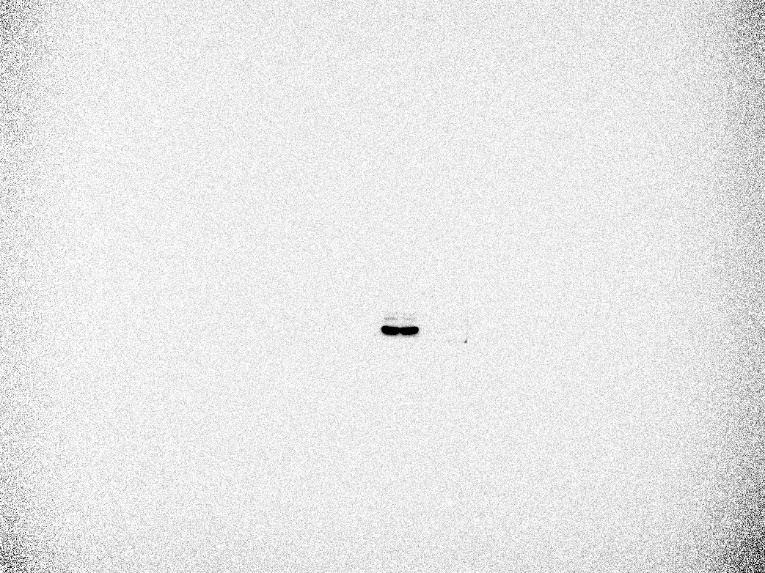


Figure 4D-MGLL:


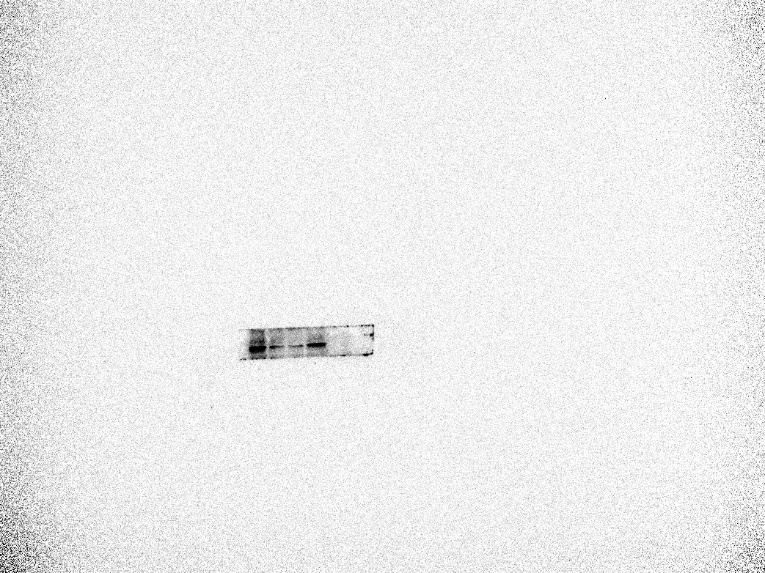


Figure 4D-GA:


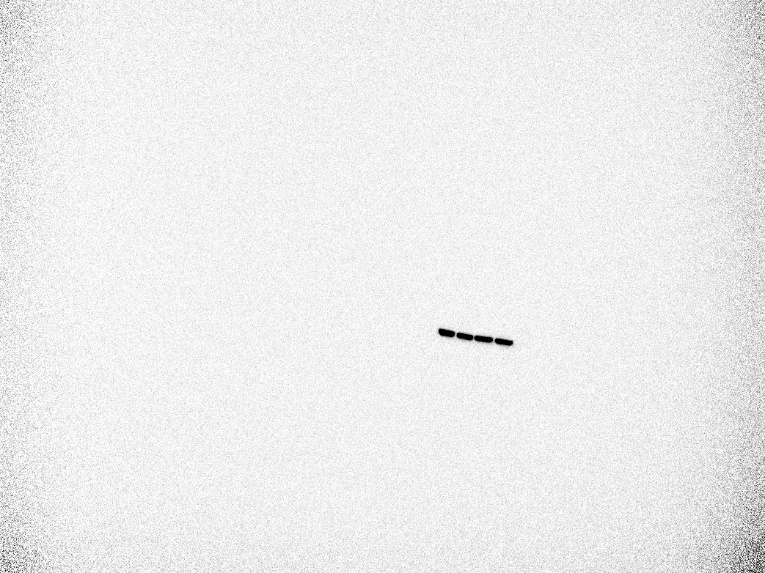


Figure 5D-YTHDF2:


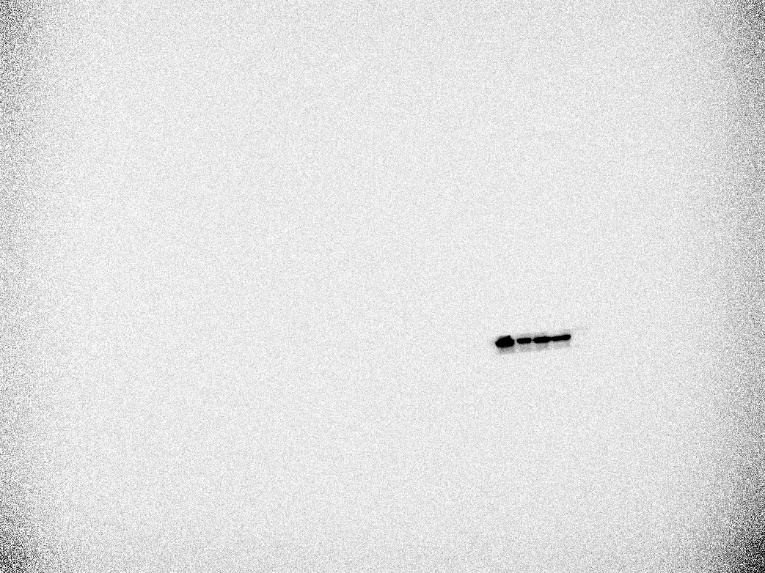


Figure 5D-GA:


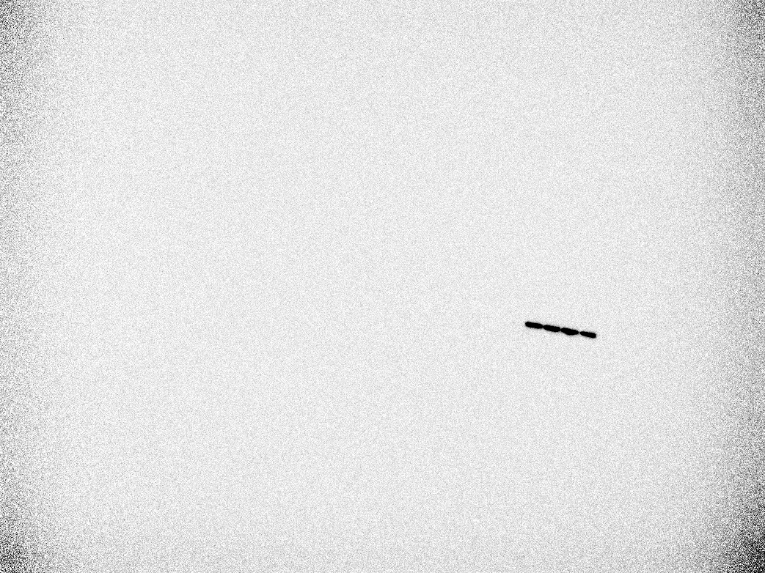


Figure 5F-MGLL:


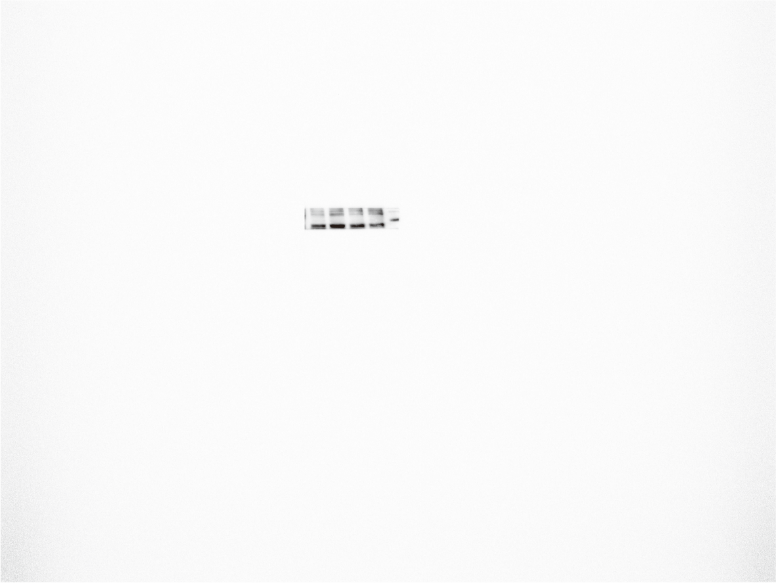


Figure 5F-GA:


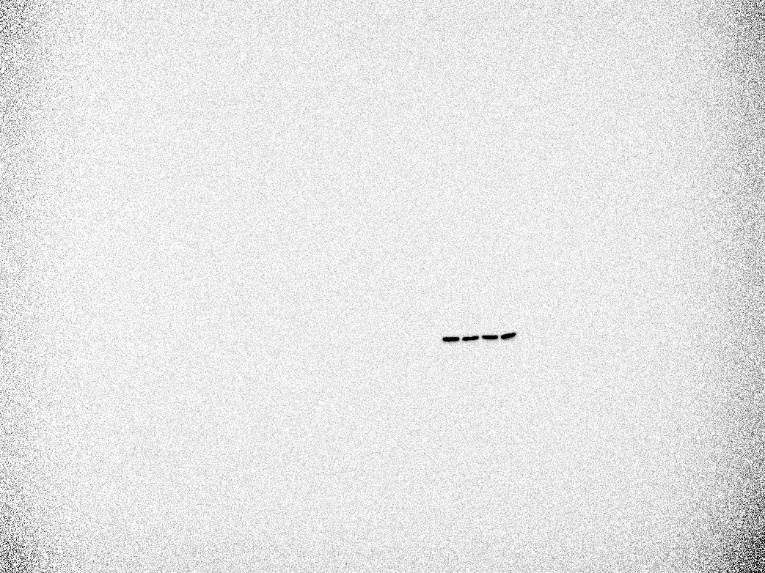


Figure 5G-YTHDF2:


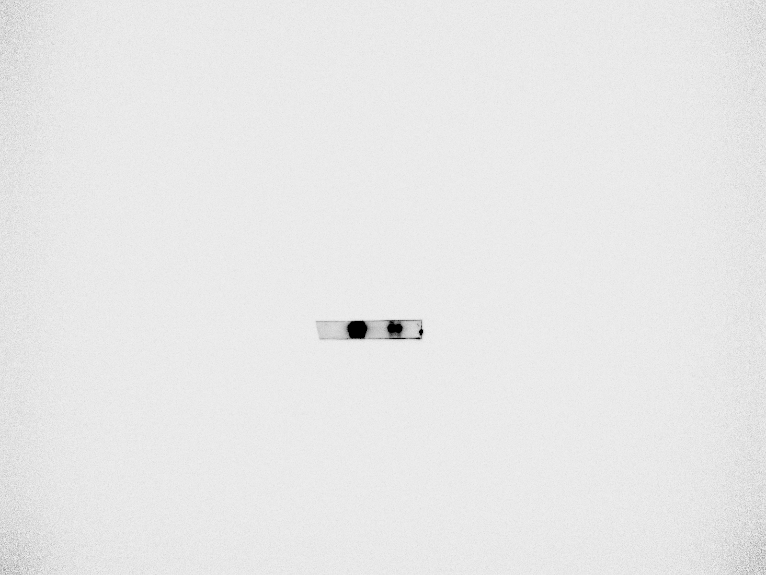


Figure 5H-YTHDF2:


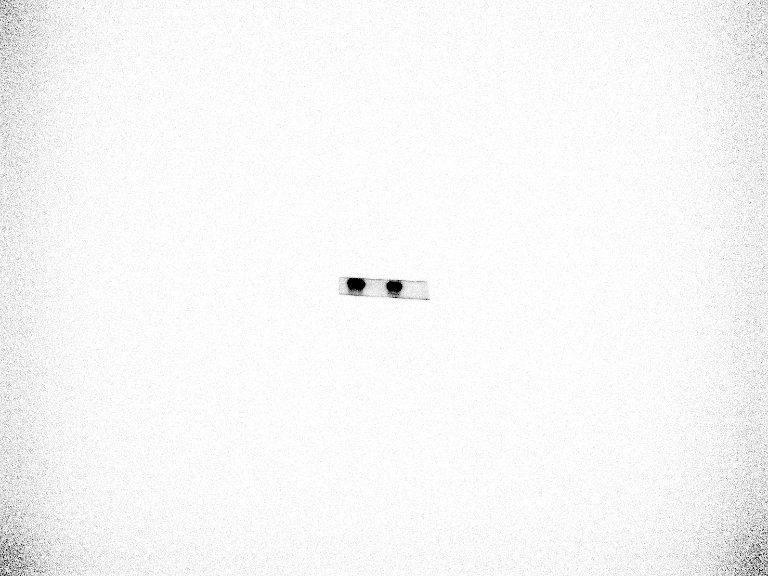


Figure S1B-YTHDC2:


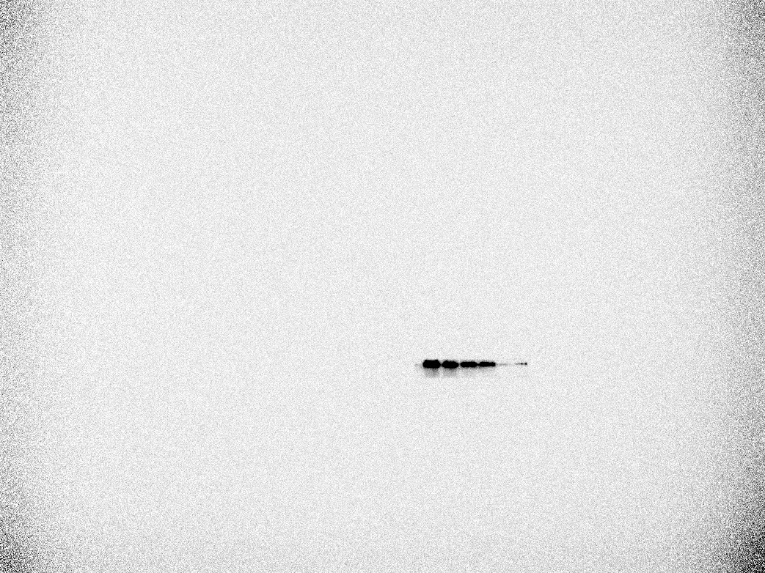


Figure S1B-GA:


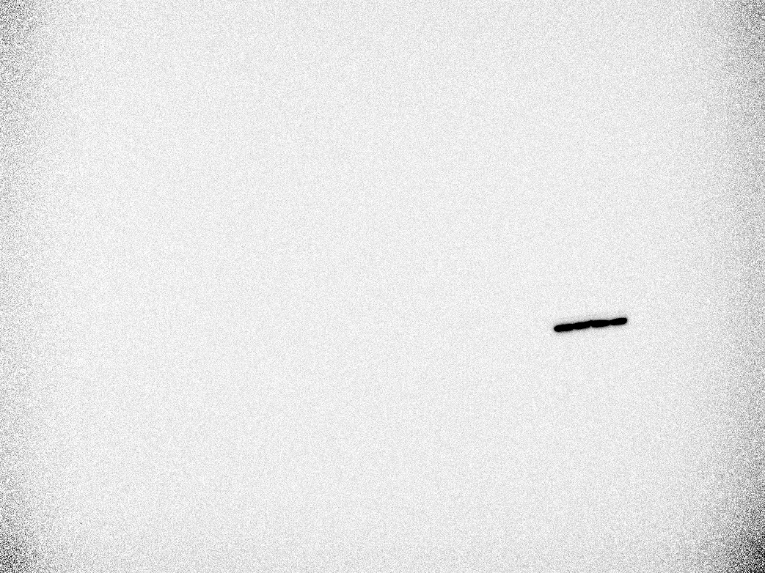


Figure S1D-YTHDF3:


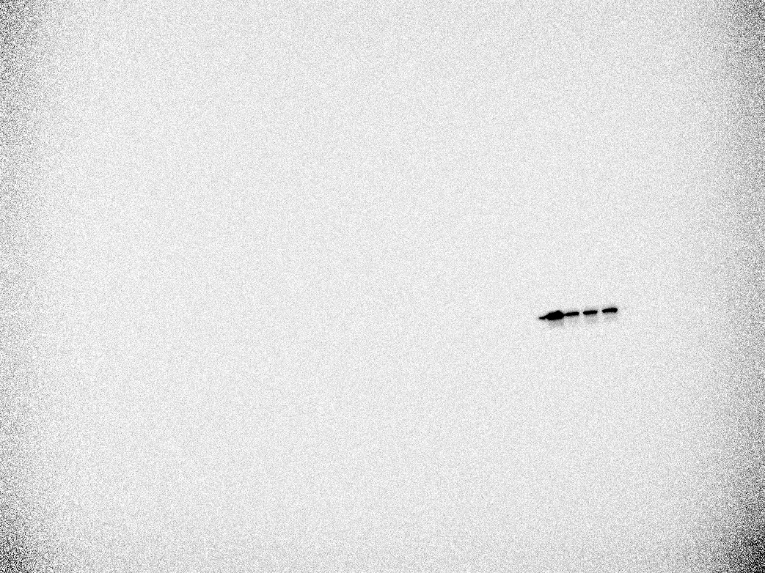


Figure S1D-GA:


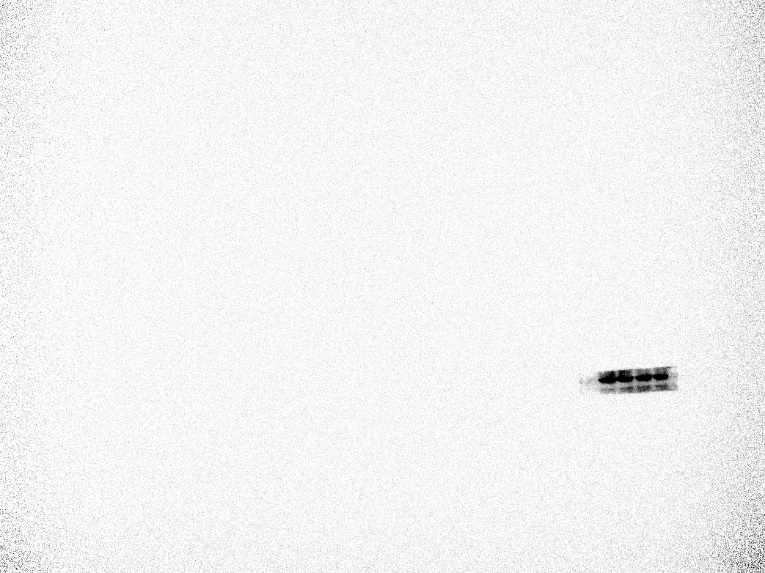

Supplement: Supplementary file 5 — Full length western blots [file 41420_2022_1149_MOESM5_ESM.docx]
